# Computational and Transcriptomic Analysis Unraveled *OsMATE34* as a Putative Anthocyanin Transporter in Black Rice (*Oryza sativa* L.) Caryopsis

**DOI:** 10.3390/genes12040583

**Published:** 2021-04-16

**Authors:** Enerand Mackon, Yafei Ma, Guibeline Charlie Jeazet Dongho Epse Mackon, Babar Usman, Yitong Zhao, Qiufeng Li, Piqing Liu

**Affiliations:** State Key Laboratory of Conservation and Utilization of Subtropical Agro-Bioresources, College of Agriculture, Guangxi University, Nanning 530005, China; breedermackon@st.gxu.edu.cn (E.M.); mayafei@st.gxu.edu.cn (Y.M.); msmackon@st.gxu.edu.cn (G.C.J.D.E.M.); babarusman119@gmail.com (B.U.); 1917301052@st.gxu.edu.cn (Y.Z.); 1717303003@st.gxu.edu.cn (Q.L.)

**Keywords:** black rice caryopsis, anthocyanin, anthoMATE, antioxidant, anthocyanin’s transport mechanism, MATE transporters, phylogenetic analysis, cyanidin-3-glucoside

## Abstract

Anthocyanin is a flavonoid compound with potential antioxidant properties beneficial to human health and sustains plant growth and development under different environmental stresses. In black rice, anthocyanin can be found in the stems, leaves, stigmas, and caryopsis. Although the anthocyanin biosynthesis in rice has been extensively studied, limited knowledge underlying the storage mechanism and transporters is available. This study undertook the complementation of computational and transcriptome analysis to decipher a potential multidrug and toxic compound extrusion (MATE) gene candidate for anthocyanin transportation in black rice caryopsis. The phylogenetic analysis showed that *OsMATE34* has the same evolutionary history and high similarities with *VvAM1*, *VvAM3*, *MtMATE2*, *SlMATE/MTP77*, *RsMATE8*, *AtFFT*, and *AtTT12* involved in anthocyanin transportation. RNA sequencing analysis in black caryopsis (Bc; Bc11, Bc18, Bc25) and white caryopsis (Wc; Wc11, Wc18, Wc25), respectively, at 11 days after flowering (DAF), 18 DAF, and 25 DAF revealed a total of 36,079 expressed genes, including 33,157 known genes and 2922 new genes. The differentially expressed genes (DEGs) showed 15,573 genes commonly expressed, with 1804 and 1412 genes uniquely expressed in Bc and Wc, respectively. Pairwise comparisons showed 821 uniquely expressed genes out of 15,272 DEGs for Wc11 vs. Bc11, 201 uniquely expressed genes out of 16,240 DEGs for Wc18 vs. Bc18, and 2263 uniquely expressed genes out of 16,240 DEGs for Wc25 vs. Bc25. Along with anthocyanin biosynthesis genes (*OsPAL*, *OsCHS*, *OsCHI*, *OsF3H*, *OsDFR*, *OsANS*, and *OsUFGT/Os3GT*), *OsMATE34* expression was significantly upregulated in all Bc but not in Wc. *OsMATE34* expression was similar to *OsGSTU34,* a transporter of anthocyanin in rice leaves. Taken together, our results highlighted *OsMATE34 (Os08g0562800)* as a candidate anthocyanin transporter in rice caryopsis. This study provides a new finding and a clue to enhance the accumulation of anthocyanin in rice caryopsis.

## 1. Introduction

Rice (*Oryza sativa* L.) is the main staple food consumed by half of the world population and almost 60% of China’s population [1]. The world’s population is expected to reach 9.7 billion by 2050; food and energy demands should be a great challenge [2]. To ensure food and nutritional security, new strategies should be adopted to increase food production and nutritional values [3]. Increasing interest in health-promoting food has significantly generated a substantial market for potential nutritionally valuable rice [4]. Pigmented or colored rice has received growing interest from many research programs due to the anthocyanin properties, particularly its potent antioxidants [5,6,7] and health benefits [3,8]. Anthocyanin is a flavonoid compound that plays a fundamental role in preventing several human diseases. Some studies suggested that anthocyanins effectively treat cardiovascular diseases, diabetes [9,10], and cancer [11,12]. Having these medicinal properties, anthocyanins are often used as alternatives for food colorants and bioactive components in nutraceutical and traditional medicine [13,14].

In black rice, anthocyanin is present in various tissues and organs such as stems, leaves, leaves sheath, ligules, apiculus, stigmas, pericarp, and endosperm in some transgenic rice. More than 18 anthocyanins and derivatives have been reported in black rice. Cyanidin-3-O-glucoside (C3G) and peonidin-3-O-glycoside (P3G) with, respectively, 65–90% and 5–28% of total anthocyanin content (TAC) are the most dominant [15]. Although anthocyanin acts as a bio-protectant molecule against reactive oxygen species (ROS) formed during respiration and photosynthesis activities [16], the antioxidant capacity of colored rice originates mainly from the seed capsule. Some studies revealed that more pigments are in the rice bran irrespective of whether the rice color is red, purple, or black [17,18]. Generally, as the rice caryopsis develops, anthocyanin accumulates mainly inside the pericarp at 7 days after flowering (DAF), thereafter inside the testa and aleurone, but not in the endosperm cells [6,19]. Therefore, some pigmented rice does not have black caryopsis or black seeds.

Anthocyanin in black rice has been extensively studied, and it is reported that its biosynthesis follows a branch of the general pathway of flavonoid compounds that begins with phenylalanine as a substrate [20,21,22]. This biosynthesis pathway is elucidated and involves structural genes (*OsPAL*, *OsCHS*, *OsCHI*, *OsF3H*, *OsDFR*, *OsANS/LDOX*, *OsUFGT/Os3GT*, etc.) and MYB-bHLH-WD40 transcription factors [23]. However, there is still a gap in the storage mechanism to make the complete puzzle. In plants, including black rice, anthocyanin is synthesized on the cell surface of the endoplasmic reticulum (ER) and stored in the vacuole via vacuolar sequestration in high concentration, which gives the intensely colored chemical structure [24,25]. The mechanism by which anthocyanin moves from the ER to the central vacuole is not clear yet. However, two models attempt to explain these mechanisms [26]. The first model is the ligandin transportations (LT), which involves ligandins that escort anthocyanin products to the vacuole and sequestrate into anthocyanic vacuolar intrusion (AVI), mostly when the concentration of anthocyanin in the cytoplasm is too high. This model believes that anthocyanin first binds to a suitable transporter (ligandin) and then diffuses through active transport until it reaches the tonoplast. The suitable transporter is glutathione-s-transferase (GST), located in the cytoplasm and associated with the ER. It acts like a binding protein that escorts anthocyanins from ER to tonoplast [27,28]. Once the anthocyanin reaches the tonoplast, it penetrates the vacuolar membrane mainly via multidrug resistance-associated proteins (MRP), representing a class of ATP-Binding Cassette (ABC) transporters [29,30]. The second model is the vesicular transport (VT) involving pre-vacuolar compartment (PVC) vesicles which drop their cargo transported to the vacuole. In this model, it is revealed that the main transporter is the multidrug and toxic compound extrusion (MATE), also called anthoMATE (AM), which accompanies PVC to the tonoplast and discard to the vacuole lumen [31]. In the VT model, AVI in vacuoles occurs as an autophagy mechanism of intact vesicles [26]. In black rice, these mechanisms are still incomplete because of the lack of information about transporters involved. Although some recent studies highlighted the implication of GST (*OsGSTU34)* and ABC (*OsMRP15*) transporters in black rice leaves [32,33], there is no supporting evidence that these transporters are involved in anthocyanin transportation in rice caryopsis. The MATE protein is a newly characterized transporter family in plants [34]. Some studies demonstrated that the MATE transporter family plays a central role in the trafficking of anthocyanin [26,28,31]. In rice, about 55 MATE genes evenly distributed throughout the 12 chromosomes in the rice genome have been deciphered. The function of MATE is very diverse and some may be involved in anthocyanin transportation [34]. However, no study has been conducted to highlight the implication of MATE transporters in rice. In the present study, we systematically investigated the MATE transporters in rice caryopsis through the combination of computational and transcriptomic analysis. Our results revealed a putative *OsMATE34* (*Os08g0562800*), a potential anthocyanin transporter gene in rice caryopsis. As new findings, these results provide a scientific foundation for understanding the anthocyanin storage mechanism in rice caryopsis. Besides, the information generated will be useful for further research to promote the high production of anthocyanin in rice improving its nutraceutical and pharmaceutical properties.

## 2. Materials and Methods

### 2.1. Plant Material

**Figure 1:**
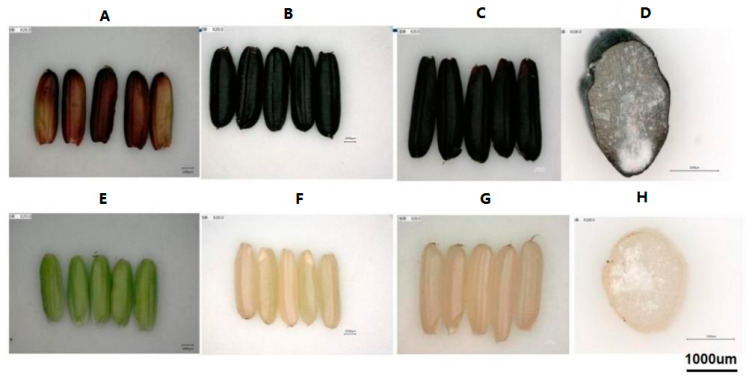
Developmental stages of black and white caryopsis; (**A**) black rice caryopsis at milk stage 11 days after flowering (Bc11); (**B**) black rice caryopsis at dough stage 18 days after flowering (Bc18); (**C**) black rice caryopsis at mature stage 25 days after flowering (Bc25); (**D**) a cross section of black caryopsis 20 days after flowering; (**E**) white rice caryopsis at milk stage 11 days after flowering (Wc11); (**F**) white rice caryopsis at dough stage 18 days after flowering (Wc18); (**G**) white rice caryopsis at mature stage 25 days after flowering (Wc25); (**H**) a cross section of white rice caryopsis 20 days after flowering.

In this study, two Indica rice cultivars with different characteristics were used: black rice cultivar “18BLN6321” with black caryopsis (Bc) and “ZiS” with white caryopsis (Wc). Rice plants were cultivated in the experimental field at Guangxi University, Nanning City, Guangxi Province (latitude: 22°49′0.01″ N, Longitude: 108°19′0.01″ E), China. This site encompasses a warm, monsoon-influenced humid semitropical climate with an annual average temperature of 21.83 °C (71.3 °F) with an average annual precipitation of 1190 mm. Rice plants were grown in the paddy field from August to November 2020. At the flowering stage, about 100 experimental plants were selected randomly and each plant carried an identification number. Samples were collected at different developmental stages; 11 DAF, 18 DAF, and 25 DAF, which represent successively milk grain, dough grain, and mature grain (Figure 1). Entire plants from both cultivars were removed from the soil and immediately transferred in the pot with soil and water, then carried to the laboratory. Rice grains were collected from about three plants, dehulled, and immediately frozen in the liquid nitrogen and stored at −80 °C until further required. At each developmental stage, two biological replicates were prepared from each cultivar named Wc11, Wc18, and Wc25 for white caryopsis, and Bc11, Bc18, and Bc25 for black caryopsis.

### 2.2. Anthocyanin Measurement

The anthocyanins were quantified between black and white caryopsis at different developmental stages using the HPLC method and three types of anthocyanin glucoside standard, namely, cyanidin-3-O-glucoside chloride, peonidin-3-O-glucoside chloride, and petunidin-3-O-glucoside chloride. Briefly, 1 g of sample was pretreated by adding 50% ethanol–water (containing 1% formic acid), then mixed using a vortex mixer for 30 s and ultrasonic for 30 min. Samples were centrifuged at 13,000 r.p.m for 30 min; the supernatants were combined and filtered using a 0.45 mm nylon filter. A 30 μL aliquot of sample was subsequently injected through the membrane of the HPLC system. The compound separations were achieved on 250 mm × 4.6 mm i.d., 5 μm reversed-phase waters XBridge C18 with 1% formic acid and acetonitrile used as mobile phase. Elution with solvent A, 1% formic acid–water, and solvent B, 1% formic acid–acetonitrile. The gradient profile for the separation of anthocyanin was 92% A–8% B (0 min). The gradient profile was subsequently changed linearly to 20%A–80%B for 20 min which was maintained for 2 min, and returned to 92% A–8% B which was maintained for 8 min. The column temperature was kept at 35 ℃; the flow rate was 0.8 mL.min^−1^ and chromatograms were recorded at 530 nm. Anthocyanins were quantified by comparing the chromatograms to external standards and the experiment was repeated three times.

### 2.3. In Silico Analysis of OsMATE in Rice

#### 2.3.1. Identification of Putative MATE Genes in Rice and other Plant and Their Sequence-Structure Analysis

A basic local alignment sequence tool for protein (BLASTP) search using the default parameters of Phytozome database (https://phy-tozome.jgi.doe.gov/pz/portal.html; accessed on 9 July 2020) and rice genome annotation project database RGAP V6.1 (http://rice.plantbiology.msu.edu/analyses_search_locus.shtml; accessed on 14 July 2020) were used to identify *Arabidopsis thaliana* MATE transporters *AtFFT, At4g25640; AtFRD3, At3g08040; AtTT12, At4g25640,* and 16 *Oryza sativa* MATE genes. The nomenclature *O. sativa* MATE (OsMATE) *OsMATE7, OsMATE34, OsMATE33, OsMATE3, OsMATE39, OsMATE16, OsMATE55,* etc. was used following Huang and co-authors. [34]. The sequences of other MATE proteins used were retrieved and can be found in the Gen-Bank/EMBL database with the following accession numbers: *Medicago truncatula MtMATE1*, ACX37118; *MtMATE2*, ADV04045.1; *Solanum lycopersicum SlMATE/MTP77*, AAQ55183; *Vitis vinifera VvMATE1*, XP_002282907; *VvMATE2*, XP_002282932; *VvAM1*, ACN91542; *VvAM3*, ACN88706; *Brassica rapa BrTT12*, ACJ36213; *Malus domestica MdMATE1*, ADO22709; *MdMATE2*, ADO22711; *Fragaria ananassa, FaTT12*: AUA60209.1; *Raphanus sativus RsMATE2*, AWK67851; *RsMATE3*, AWK67852; *RsMATE7*, AWK67856; *RsMATE8*, AWK67857;

The physicochemical properties of MATE proteins were submitted and computed to identify the molecular weight (MW), the number of amino acids (A.A), the isoelectric point (PI), the aliphatic index (AI), the instability index (I.I), and the grand average of hydropathicity index (GRAVY) using the ExPASy Compute pI/Mw tool (http://https://web.expasy.org/cgi-bin/protparam; accessed on 30 August 2021).

#### 2.3.2. Phylogenetic Analysis and Gene Structure

ClustalW (http://clustalw.ddbj.nig.ac.jp/top-e.html; accessed on 26 July 2020) was used to align the 38 full-length MATE proteins. Subsequently, the rooted and unrooted phylogenetic trees were constructed using MEGA X_10.1.8 with the maximum likelihood (ML) algorithm and 1000 bootstraps to infer the evolutionary history based on the Jones–Taylor–Thornton (JTT) matrix-based model. By applying Neighbor-Join and BioNJalgorithms to a matrix of pairwise distances using a JTT model, we automatically obtained the initial tree(s) for heuristic search. The result was viewed and then edited using evolview [35]. The percentage of trees in which the associated taxa clustered together is shown next to the branches. The sequence homology, similarity, and identity were analyzed through a multiple sequence alignment using Clustal Omega multiple sequence analysis (https://www.ebi.ac.uk/Tools/msa/clustalo/; accessed on 21 August 2020). The structure of gene transcripts with the exons and introns was retrieved through different plant’s online databases and mapped. The conserved protein motifs of MATEs were analyzed using the multiple expectation maximization for motif elicitation (MEME) online program (http://meme-suite.org/; accessed on 14 August 2020) [36] with default settings and a maximum of 12 motifs. Three-dimensional protein structure for various MATE protein (*OsMATE34, OsMATE7, VvAM1, VvAM3, AtFFT, SlMATE77, MtMATE2, RsMATE8*) was constructed by using Phyre2 software (http://www.sbg.bio.ic.uk/phyre2; accessed on 25 January 2021) with fold recognition end homology modeling for the identification of secondary structure attributes. The numbers of transmembrane helices and subcellular localization of the MATE proteins were predicted by transmembrane helices hidden Markov model (TMHMM) server (www.cbs.dtu.dk/services/TMHMM2.0/; accessed on 30 August 2021) and WoLF PSORT (http://www.genscript.com/wolf-psort.html; accessed on 8 September 2021), respectively, with default parameters.

### 2.4. Transcriptome Analysis

#### 2.4.1. RNA Isolation, Library Preparation, and Illumina Hiseq Xten/Nova seq 6000 Sequencing for Transcriptome Analysis

Total RNA was isolated from each sample representing the white and black caryopsis separately using the TRIzol^®^ Reagent kit (Invitrogen, Carlsbad, CA, USA) according to the manufacturer’s instructions. The concentration and purity were analyzed using NanoDrop2000 spectrophotometer and RNA integrity was detected by agarose gel electrophoresis, the Agilent 2100 measured the RIN value. The cDNA library was generated using the TruSeqTM RNA sample preparation kit (San Diego, IL, CA). Briefly, the polyA selection method by oligo (dT)-attached magnetic beads was used to isolate and enrich mRNA from total RNA. Then, the fragmentation of mRNA into a small sequence of ~300 bp length was carried out by adding fragmentation buffer. Fragmented mRNA templates were transcribed into the first-strand cDNA followed by the synthesis of second-stranded cDNA by using reverse transcriptase (Invitrogen, CA, USA) and random hexamer primers (Illumina) to form a stable double-stranded structure. The cDNA structure sticky end was end-repaired followed by adenylation of the 3′ end poly (A) tail and ligation of index adaptors to prepare for hybridization. After that, the amplification of purified cDNA (using Phusion DNA polymerase by NEB) was performed by polymerase chain reactions (PCR) for 15 cycles. PCR products were separated on 2% low-range ultra-agarose (Certified Low Range Ultra Agarose, Bio-Rad) and quantified by TBS380 Picogreen, Invitrogen. Amplified fragments were subjected to deep sequencing by Illumina Hiseq Xten/NovaSeq 6000 sequencer platform, performed by Shanghai Majorbio Bio-pharm Technology Corporation (Shanghai, China).

#### 2.4.2. Data Filtering and Mapping of Reads

Before data analysis, a quality control of raw data was carried out and the raw reads were filtered to obtain high-quality reads (clean data) to ensure the smooth progress of subsequent analysis. High-quality reads were assembled using SeqPrep (http://github.com/jstjohn/SeqPrep; accessed on 28 November 2020) and Sickle (http://github.com/najoshi/sickle; accessed on 28 November 2020) software with default parameters. This included removing of reads linker sequence, trimming the base with low quality (less than 30) at the end of the 3′ end sequence, reads having more than 10% N ratio, and discarding the adaptor and sequence with final length less than 50 bp after quality trimming. Afterward, clean reads were separately aligned to the *O. sativa japonica* ref genome IRGSP-1.0 ref_genome (http://plants.ensembl.org/Oryza_sativa/Info/Index; accessed on 5 December 2020) to obtain mapped data (reads) using HISAT2 (http://ccb.jhu.edu/software/hisat2/index.shtml; accessed on 10 December 2020) or TopHat2 (http://tophat.cbcb.umd.edu/; accessed on 10 December 2020) [37] software and assembled by StringTie (https://ccb.jhu.edu/software/stringtie/index.shtml?t=example; accessed on 16 December 2020) in a reference-based approach [38].

#### 2.4.3. Differential Expression Functional Enrichment Analysis

For the analysis of RNA-Seq data, differentially expressed genes sequencing (DEGSeq) was performed to identify the differentially expressed genes (DEGs) in rice caryopsis affected by the accumulation of anthocyanin between different samples. Gene abundances were quantified by RNA sequence by expectation maximization (RSEM) (http://deweylab.biostat.wisc.edu/rsem/; accessed on 20 December 2020) [39]. The TPM referring to transcripts per million reads methods was used to normalize the expression value of reads for all 12 samples. The analysis of differential expression between samples was performed using the DESeq2 [40], and pairwise comparison was applied. Our testing criterion for screening DEGs was based on multiples hypotheses with the *p*-value threshold, false discovery rate (FDR) (≤0.05), and (log 2 FoldChange ≥ 1) considered as significantly different. Gene function was annotated based on database non-redundant databases, Swiss-Prot, cluster of orthologous groups databases, gene ontology, and the Kyoto Encyclopedia of Genes and Genomes Pathway database to get functional annotation of DEGs e-value cut-off E < 1 × 10^−5^. GO enrichment analysis of transcript in the gene set was performed using the software Goatools 0.6.5 (https://github.com/tanghaibao/GOatools; accessed on 5 January 2021) [41] and considered significantly enriched when *p*-value (FDR) ≤ 0.05.

### 2.5. Expression Analysis of Putative Anthocyanin OsMATE Genes by Reverse Transcription Quantitative PCR (RT-qPCR) 

To evaluate the gene expression, we performed RT-qPCR. The total RNA was isolated from rice caryopsis using the TRIzol^®^ Reagent kit (Invitrogen, Carlsbad, CA, USA) according to the manufacturer’s instructions. We chose four candidate genes differentially expressed in black and white rice caryopsis including two OsMATEs (*OsMATE34* and *OsMATE3*), one GST *(OsGSTU34*), and one MRP (*OsMRP15*). Rice *OsActin1* was used as an internal reference gene to normalize the gene expression level. The primer sequences listed in Table S1 were retrieved from https://biodb.swu.edu.cn/qprimerdb/best-primers-ss; accessed on 4 February 2021. First-strand full-length cDNAs were synthesized from 2 µg of total RNA using the primeScript^TM^ RT reagent kit with gDNA eraser (RR047A, Takara, Japan) according to the manufacturer’s instruction. RT-qPCR was carried out using the ChamQ^TM^ SYBR qPCR Master Mix (Q311-01, Vazyme, China) on the LightCycler 480II (Roche) according to the manufacturer’s instructions. Three biological repeats were used for each treatment (Bc11, Bc18, and Bc25) and control (Wc11, Wc18, and Wc25) for gene expression profiles. The reaction was adjusted following the thermal cycling conditions as initial denaturing temperature, 95 °C for 30 s, followed by 40 cycles and each cycle consisted of 95 °C for 5 s and 60 °C for 20 s. The gene expression level was calculated by the 2^−^^ΔΔ^^Ct^ calculation method.

## 3. Results

### 3.1. Anthocyanin Quantification at Different Developmental Stages

We performed high-performance liquid chromatography (HPLC) to analyze the two major rice anthocyanins (C3G and P3G) and one minor anthocyanin (Petunidin-3-O-glucoside, Pt3G) in black and white caryopsis. Our results showed that Pt3G was not detected in both black and white caryopsis, while C3G and P3G were detected only in black caryopsis (Table 1). The amount of anthocyanin varied at different developmental stages. The peak was attained at 18 DAF in sample Bc18. At this stage, the concentration of C3G and P3G was 4206.91 ± 18 µg/g of fresh weight (FW) and 295.33 ± 4 µg/g FW, respectively. The concentration of C3G varies between 91 and 93% of TAC while P3G was approximately 7–9% of TAC (Figure S1).

### 3.2. Phylogenetic Tree Analysis of MATE Proteins and Deduction of Putative OsMATE Gene for Anthocyanin Transportation

**Figure 2:**
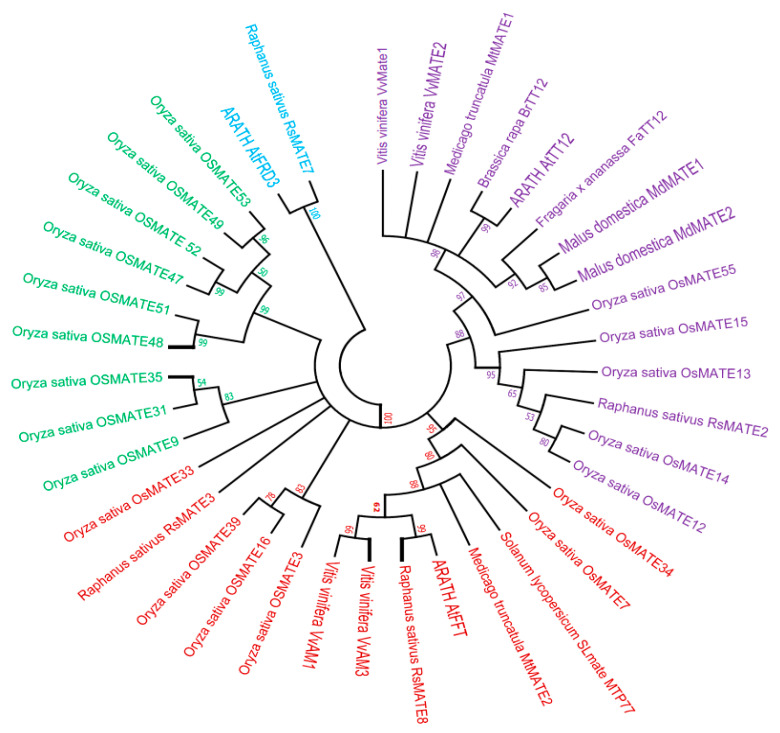
Molecular phylogenetic tree of OsMATEs proteins. Each color represents one cluster (CL): cluster1 (CL1), blue color; cluster2 (CL2), green color; cluster3 (CL3), red color; cluster4 (CL4), violet color.

From 55 MATE proteins identified in *O. sativa*, we selected 20 MATE transporters [34] and 18 other MATE transporters with well-known functions. The phylogeny relationships suggested that the function of MATE proteins in rice could be deduced based on the known function of MATE transporters in other plants. Homologous sequences having similar structures were found to have similar functions as well [42]. To establish the evolutionary relationships between selected OsMATEs and other MATE-like proteins, the phylogenetic trees were generated after the alignment of all 38 full-length amino acid sequences. Overall, the tree showed four different clades (CL) named CL1, CL2, CL3, and CL4 (Figure 2). Remarkably, the proteins known as anthocyanin transporters were separated from others having different functions. The CL1 did not contain rice MATE transporters and consisted of MATE proteins previously identified in *R. sativus (RsMATE7)* and *A. thaliana* (*AtFRD3*); *AtFRD3* is involved in iron homeostasis [43]. In terms of evolution, CL1 appears as the root ancestor of all MATE transporters of the tree. The CL2 consisted of two sub-groups, CL2-1 and CL2-2, all found in rice. The function of these MATEs has been inferred from the function of *OsMATE9* [34] previously identified as *OsMATE1* and involved in the development of rice, especially fertility, disease, and stress tolerance [44]. The CL3 split into two sub-groups: CL3-1 and CL3-2. The CL3-1 comprised four rice MATE proteins—*OsMATE33, OsMATE39, OsMATE16, OsMATE3*—and one radish MATE transporter protein (*RsMATE3*) previously presumed to transport anthocyanin in radish [45]. The sub-group CL3-2 comprised two rice MATE proteins (*OsMATE7* and *OsMATE34*) and six characterized transporters of various anthocyanin types such as acylated anthocyanin *VvAM1* and *VvAM3* from *V. vinifera* [31,46]; flavone glycoside or malonylated anthocyanin *MtMATE2* from *M. truncatula* [47]; and three others were not specific including *SlMATE* or *MTP77* from *S. lycopersicum* [48], *RsMATE8* from *R. sativus* [45], and *AtFFT* from *A. thaliana* [49] associated with the transport and accumulation of glycosylated anthocyanin inside the vacuole. CL4 consisted of two sub-groups: CL4-1 and CL4-2. CL4-1 is composed of five rice MATE genes previously assumed to be related to vacuolar transport and flavonoid or alkaloid accumulation in the plant [34]. The subgroup CL4-2 was constituted oby one rice MATE *OsMATE55* clustered with eight well-identified proanthocyanidin MATE-like transporters in several plants including *AtTT12* from *A. thaliana* [50], *BrTT12* from *Brassica rapa* [51], *VvMATE1* and *VvMATE2* from *V. vinifera* [52], *MtMATE1* from *M. truncatula* [53], *MdMATE1* and *MdMATE2* from *Malus domestica* [54], and *FaTT12* from *F. ananassa* [55]. These MATEs were involved in mediating the transport of proanthocyanidins and the transport of flavonoids and epicatechin-3’-O-glucoside into the vacuole. The phylogenetic scrutiny is one of the fastest, simplest, and relatively most precise ways to predict gene function that can be subsequently prioritized for further practical confirmation [56]. Previous studies have provided strong evidence for function prediction of gene families based on phylogenetic analysis, including MATE transporters [34,56,57,58]. This analysis suggested that *OsMATE33, OsMATE39, OsMATE16, OsMATE3, OsMATE7*, and *OsMATE34* could be associated with anthocyanin transportation and were selected as candidate proteins, while *OsMATE55* could be involved in transporting proanthocyanidin in rice and warrant further analysis.

### 3.3. Molecular Characteristics and Structure of Putative Anthocyanin MATE Protein

The putative OsMATE proteins are composed of 363–522 residues with predicted molar masses of approximately 38.74–56.17 kDa and theoretical PI of 4.72–9.58, aliphatic indices from 110.08–119.94, and grand average hydropathicity index of 0.528–0.806. Compared to other MATE-like proteins, these values are similar (Table S2). However, high similarities were particularly identified between some OsMATE and other anthocyanin MATE proteins studied such as *OsMATE34* with *MtMATE2* and *VvAM1, OsMATE33,* and *VvAM3*. *OsMATE7* had the lowest values for these indices, meaning that its structure might be different from other MATE. The instability index of all OsMATE proteins and other known anthocyanin MATE proteins was found to be less than 40, predicting that these proteins are stable. The prediction of the secondary structure of OsMATE by the self-optimized prediction method from alignment (SOPMA) showed that these *O. sativa* MATEs were mainly made of α-helices (59.20–64.46%), distributed with random coils (17.46–22.31%), extended strands (13.84–16.56%), and β turns (3.31–4.02%) (Figure S2). Transmembrane domains predicted by hidden Markov model topology (HMMTOP) revealed that OsMATEs have variant putative transmembrane (TM) segments ranging from 6 (*OsMATE7*), 10 (*OsMATE39*), 11 (*OsMATE3*), and 12 (*OsMATE16, OsMATE33, OsMATE34*) (Figure S3). The TM structure of *OsMATE33* and *OsMATE34* was similar to *MtMATE2* and *AtFFT*; that of *OsMATE16* was similar to *RsMATE8* and *VvAM3*, and *OsMATE3* was similar to *VvAM1* and *SlMATE*. Reportedly, the MATE family consists of a unique topology predicted to have 10–12 TM helices with long, cytoplasmatic C and N termini [59].

**Figure 3:**
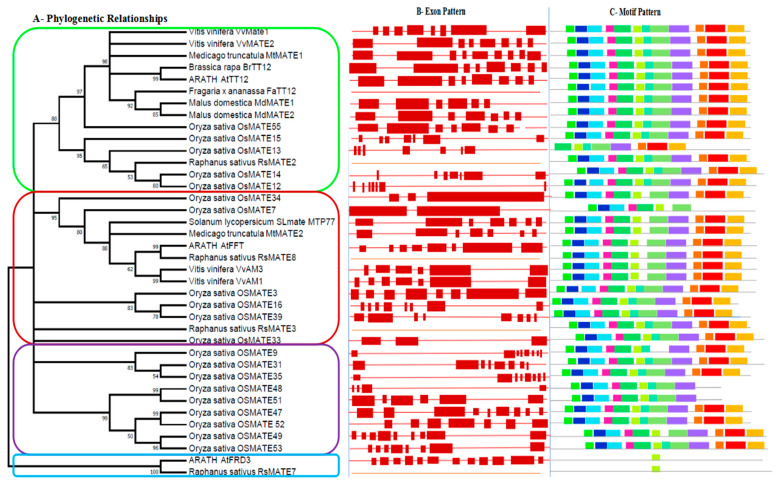
Phylogenetic relationships and domain compositions of the MATE proteins. (**A**) The unrooted phylogenetic tree was constructed with 1000 bootstrap replicates based on multiple alignments of 38 MATE amino acid sequences. The four major subgroups are marked with a different colored line. Each color represents one cluster (CL): cluster1 (CL1), blue color; cluster2 (CL2), green color; cluster3 (CL3), red color; cluster4 (CL4), violet color. (**B**) Exon pattern, red boxes represent exons while lines represent introns. (**C**) The conserved motifs in the MATE proteins were identified using MEME, gray lines represent the non-conserved sequences. Each motif is indicated by a colored box numbered at the bottom. The length of the motifs in each protein is exhibited proportionally. Twelve motifs are illustrated using different colored boxes to get more information about the motif sequence, refer to Table S3.

The exon–intron pattern of the coding sequence was explored and plotted with the phylogenetic tree (Figure 3A) to provide some insight into the evolution of gene structure (Figure 3B). The result revealed some variation of exons and introns number and length in different branches of MATE precisely in clade CL3, which harbored anthocyanin MATE-like transporter proteins. In this clade, the number of exons and introns range from 2–8 and 1–7, respectively, and all had untranslated regions. *OsMATE33*, *OsMATE34,* and *OsMATE7* had 3,3,2 exons and 2,2,1 introns, respectively, which were fewer compared to others. They were also connected with the same common direct ancestor. The number of exons and introns gradually increases with the increase of nod, and in subclade CL3-1, *OsMATE3, OsMATE16,* and *OsMATE39* had eight exons and even introns with untranslated region similar to *AtFFT* and relatively the same to *SlMATE*, *MtMATE2*, *VvAM1,* and *VvAM1*.

To confirm the similarities between sequences observed, the protein motifs patterns were analyzed by MEME (Figure 3C). The result showed that the sequences and types of protein motifs were similar within the same clade. The motif structure is highly similar in the CL3 clade except for *OsMATE7* with only eight protein motifs. Furthermore, the subclade CL3-2 compared to CL3-1 showed the loss of the protein motif 7 during evolution. Thus, CL3-1 had 12 protein motifs while CL3-2 had 11, and the MATE proteins *OsMATE7* were significantly different from other proteins with few motifs. This remarkable difference in MATE protein structures might be due to functional diversities that occurred during the evolution.

Homology sequences were analyzed through a multiple sequence alignment of six putative OsMATE amino acid sequences and selected MATE-like proteins associated with anthocyanin transportation in other plants. The result showed that OsMATE shared 31–55% and 40–60% similarity and identity, respectively, in their amino acid sequence with other MATEs. *OsMATE34* and *OsMATE7* had the highest identity with *MTP77/SlMATE*, *MtMATE2, AtFFT, RsMATE8, VvAM1,* and *VvAM3* ranging between 52.66 and 61% whereas *OsMATE3, OsMATE16* and *OsMATE33, OsMATE39* showed high identity with *RsMATE3* ranging from 47.08 to 51.58% (Table S4). Strikingly, *OsMATE33* and *OsMATE7* exhibited, respectively, 57% and 61.39% identity with *AtFFT* amino sequence associated with anthocyanin transportation in *Arabidopsis thaliana* [49].

The subcellular localization of OsMATEs predicted that these proteins were localized in different organelles such as plasma membrane, vacuole, endoplasmic reticulum, Golgi apparatus, chloroplast, and mitochondria. However, the result showed some differences in the localization pattern. Like *SlMATE* and *MtMATE2*, *OsMATE34* and *OsMATE39* were mainly found in the plasma membrane and vacuole; *OsMATE3* and *OsMATE33* in the plasma membrane similar to AtFFT and *VvAM1; OsMATE16* was found inside the vacuole and *OsMATE7* in the plasma membrane, endoplasmic reticulum, and cytoplasm (Table S5). Signal peptides often guided proteins to their target membranes; however, these proteins seemed to have no signal peptides in the N-terminal according to signal. Sometimes peptides are difficult to identify because there is some possibility that they do not have strictly conserved amino acid sequences; thus, the first transmembrane domain which biochemically resembles a signal sequence can play a signal function for translocation.

### 3.4. Transcriptome Sequencing and De Novo Assembly

**Figure 4:**
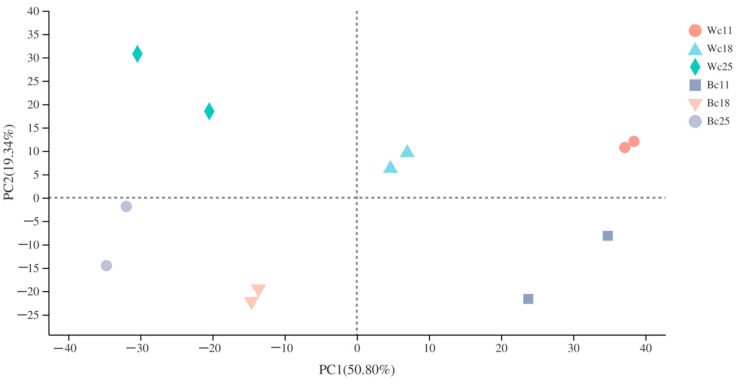
Principal component analysis of replicates samples at different developmental stages. Wc11 = White caryopsis, 11 days after flowering; Wc18 = White caryopsis, 18 days after flowering; Wc25 = White caryopsis, 25 days after flowering; Bc11 = Black caryopsis, 11 days after flowering; Bc18 = Black caryopsis, 18 days after flowering; Bc25 = Black caryopsis, 25 days after flowering. The distance of each sample point represents the distance of the sample. The closer the distance, the higher the similarity between the samples. The horizontal axis represents the contribution of principal component 1 (PC1) to the distinguished sample in the two-dimensional graph, and the vertical axis represents the contribution of principal component 2 (PC2) to the distinguished sample in the two-dimensional graphs.

We constructed a total of 12 cDNA libraries from twelve samples (six for black caryopsis and six for white caryopsis) collected at three different rice developmental stages—11 DAF, 18 DAF, and 25 DAF—for sequencing using the high-throughput Illumina Hiseq Xten/Nova sequencing for transcriptome analysis. After sequencing, 44.23–57.72 million (M) items of raw data were obtained. Data filtration further generated 43.81–57.13 million (M) paired-end clean reads from each sample with Q30 percentage > 95.8%, GC percentage > 51%, and additional analysis showed a Pearson correlation coefficient of each of two replicates sample greater than 0.96. These data indicated a great similarity between the two biological replicated samples and the good quality of the sequencing result. The percentages of mapped reads generated after mapping the clean data of each sample to the rice reference genome were comprised between 92–95% (Table 2). Subsequently, the principal component analysis showed that replicated samples were relatively close, while the distance between different cultivars was relatively high (Figure 4). This result highlighted the genetic similarity and difference between samples and cultivars respectively at different stages.

### 3.5. Identification of Differentially Expressed Genes (DEGs)

**Figure 5:**
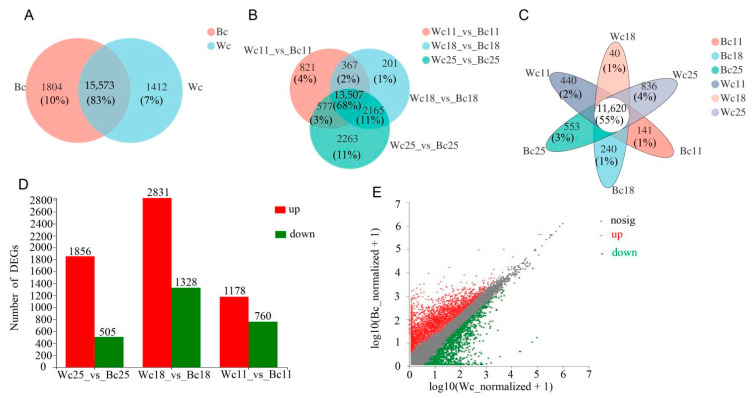
Comparison of transcript and gene abundance in black and white caryopsis. (**A**) Venn diagram of commonly expressed and unique genes in black and white rice caryopsis. (**B**) Venn diagram of the differentially expressed genes (DEGs) in the black and white caryopsis at different developmental stages. (**C**) Venn diagram of commonly expressed and uniquely expressed genes in a different sample at different stages. (**D**) The number of DEGs between black and white caryopsis at 25 DAF, 18 DAF, and 11 DAF. The red bars denote the upregulated genes and the green indicate the downregulated genes. (**E**) Scatter diagram with the abscissa and ordinate, respectively, represents the expression levels of genes in white and black caryopsis where the value of the abscissa and the ordinate were logarithmicized and each point represents a specific gene. Red dots show significantly upregulated, green dots represent genes that were significantly downregulated, and gray dots non-significantly different genes. The closer the point is to 0, the lower the expression level; the greater the deviation from the diagonal line, the greater the difference in expression of a gene between the two samples.

Gene abundances were quantified by RSEM (http://deweylab.biostat.wisc.edu/rsem/), and a total of 36,079 expressed genes were detected from the twelve samples in this analysis, including 33,157 known genes and 2922 new genes; 59,966 expressed transcripts, including 38,802 known transcripts and 21,164 new transcripts were identified. DEGs were essentially analyzed using Deseq2 log2FoldChange ≥ 1 and the *p*-value cut-off (FDR ≤ 0.05). Further pairwise comparison of the number of the expressed gene between the caryopsis of two cultivars at different developmental stages showed that 15,573 genes were commonly expressed in both black and white caryopsis, while 1804 and 1412 genes were uniquely expressed in black and white caryopsis, respectively (Figure 5A). We conducted three comparisons across rice caryopsis at different developmental stages, the result showed that 821 uniquely expressed genes out of 15,272 DEGs for Wc11 vs. Bc11, 201 uniquely expressed genes out of 16,240 DEGs for Wc18 vs. Bc18, and 2263 uniquely expressed genes out of 16,240 DEGs for Wc25 vs. Bc25 (Figure 5B). Subsequent analysis showed that in both caryopsis types, uniquely expressed genes were higher at 25 DAF with 836 and 553 genes in Wc25 and Bc25, respectively (Figure 5C). The further comparison made based on two rice cultivars Wc vs. Bc showed that upregulated genes were higher than downregulated genes at all stages (Figure 5D) and the number of up-regulated genes was higher in black caryopsis (Figure 5E). 

### 3.6. Functional Annotation and Enrichment Analysis of DEGs

**Figure 6:**
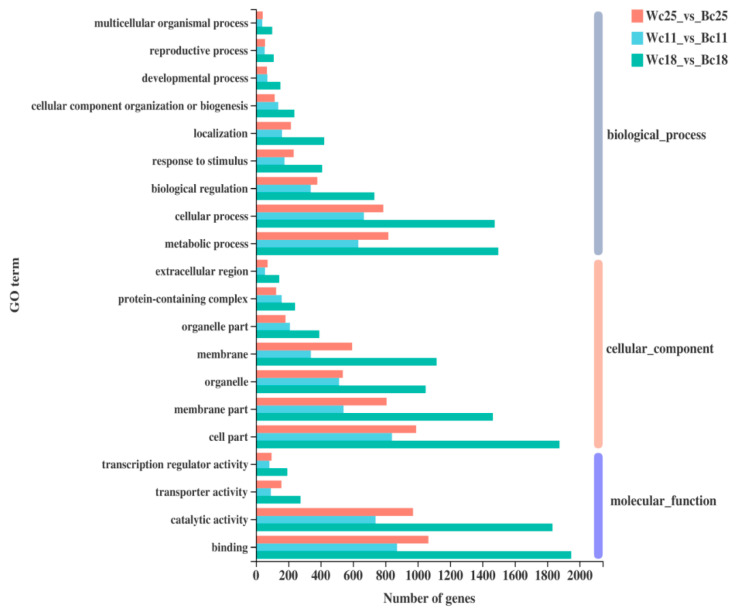
Gene ontology analysis of DEGs; results are shown in three categories: Biological Process (gray), Cellular Component (orange), and Molecular Function (violet); the left axis represents the number of genes of each term, while the *Y*-axis represents the GO term; different colored bars represent different comparisons.

To characterize the main biological functions of DEGs, we carried out gene function annotation of all transcripts and expressed genes in rice caryopsis. These transcripts were annotated by non-redundant (Nr) databases, Swiss-Prot, the cluster of orthologous groups databases (COG), gene ontology (GO), and the Kyoto Encyclopedia of Genes and Genomes pathway database (KEGG). We further performed GO level 2 on three sets of DEGs (Wc11 vs. Bc11, Wc18 vs. Bc18, and Wc25 vs. Bc25) and three GO categories, namely, biological process, cellular component, and molecular function, were revealed (Figure 6). In the category biological process, metabolic process, cellular process, and biological regulation were particularly high. In the category cellular component, cell part, membrane part, and organelle were highest, and in the category molecular function high level of the gene showed for binding and catalytic activity. 

**Figure 7:**
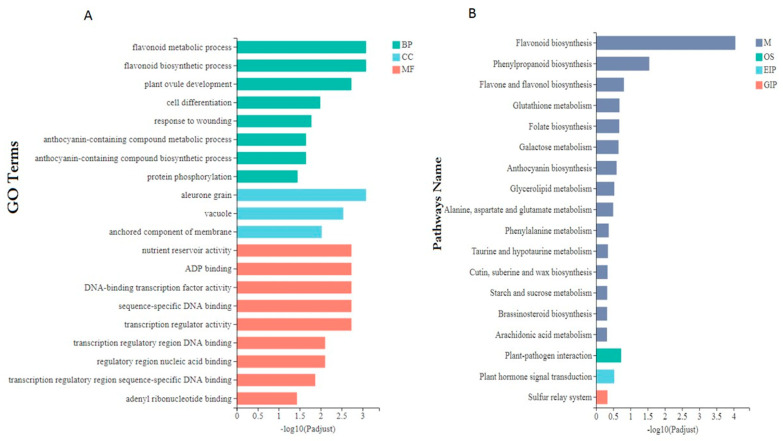
GO and KEGG enrichment: (**A**) Bar diagram showing GO enrichment in Bc vs. Wc; MF indicates the category molecular function; CC, the category cellular component; BP, the category biological process. (**B**) Histogram showing KEGG enrichment in Bc vs. Wc; the ordinate represents the KEGG pathway and the abscissa represents the significance level of enrichment under *p*-adjust < 0.5, which corresponds to the height of the column. The smaller the FDR and the greater the −log10 (FDR) value, the more significantly enriched the KEGG pathway. Different colors indicate 4 enriched branches of the KEGG metabolic pathway: genetic information processing (GIP), environmental information processing (EIP), biological systems (OS), and metabolism (M).

To predict the biological function of different DEG sets, we investigated the GO enrichment in Wc vs. Bc. The results indicated that in the category biological process, flavonoid metabolic (GO:0009812) and biosynthesis (GO:0009813) processes were particularly enriched, while in the category cellular component, aleurone grain (GO:0033095) and vacuole (GO:0005773) were enriched. In metabolic function, the sub-categories nutrient reservoir activity (GO:0045735), ADP binding (GO:0003677), DNA binding transcription regulator activity (GO:0003700), transcription regulator activity (GO:0140110), and a sequence-specific DNA activity (GO:0043565) were enriched (Figure 7A). Meanwhile, all DEGs were suggested to KEGG analysis and the result showed that carbohydrate metabolism (62 genes over 854) and biosynthesis of secondary metabolite process (66 genes) were high in all groups of comparison and mainly Wc vs. Bc (Supplementary file 1). The KEGG enrichment indicated that metabolism pathways were predominant and subsequent analysis revealed that flavonoid biosynthesis pathways (KEGG: map 00941) and phenylpropanoid pathway (KEGG: map 00940) were particularly enriched (Figure 7B). 

### 3.7. Expression of Anthocyanin Biosynthesis Genes and Transporters Genes in Rice Caryopsis

**Figure 8:**
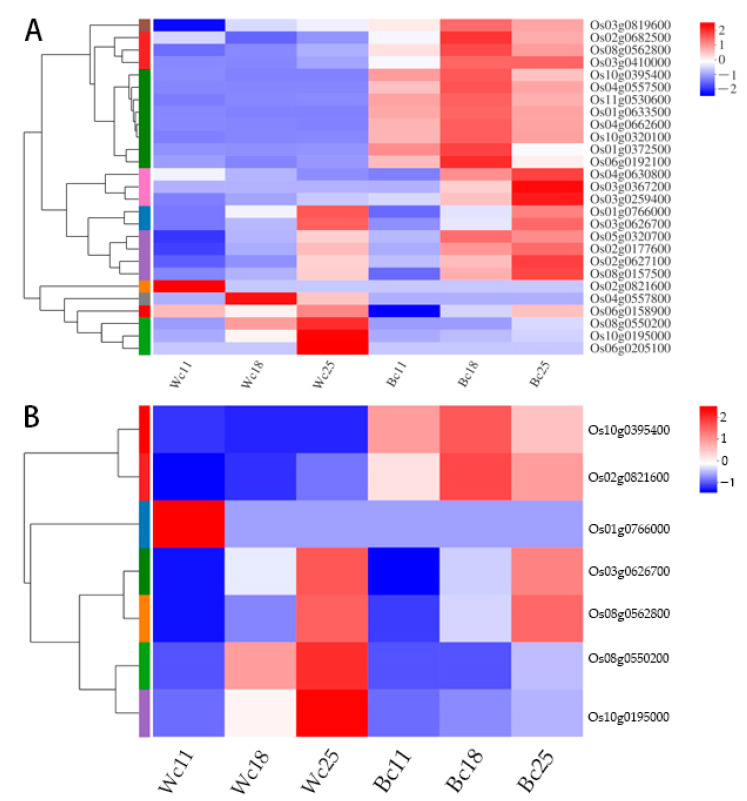
Heatmap showing expression profile and cluster analysis of DEGs in different samples: (**A**) Expression profile of twenty-seven anthocyanin biosynthesis DEGs including putative transporters. (**B**) Heatmap representation and hierarchical clustering of the transporters genes of various samples in black and white caryopsis. The transcript data of six samples from rice caryopsis were used to reconstruct the expression patterns of genes. The clear boxes indicate that the transcript abundance is zero. The bar at the right side of the heat map represents the relative expression values; values below 0 represent downregulated expression and values above 0 represent upregulated expression.

To further understand the implication of anthocyanin biosynthesis genes, and mainly transporter genes, in rice caryopsis, we carried out different pairwise comparison analysis Wc11 vs. Bc11, Wc18 vs. Bc18, and Wc25 vs. Bc25 of twenty-seven rice genes including fourteen anthocyanin structural genes, five regulatory genes, two well-known transporter genes, and six unknown genes putative transporters. The expression pattern was different. All fourteen structural genes were upregulated, three out of five structural genes were upregulated in Wc11 vs. Bc11, Wc18 vs. Bc18, and Wc25 vs. Bc25 with most of them highly significant (Supplementary file 2). These genes have been previously identified as key genes in anthocyanin biosynthesis pathways. The heatmap showed that most of these genes are mainly upregulated in black caryopsis and downregulated in white caryopsis (Figure 8A). The analysis of transporters’ genes indicated that among two known transporters (*OsGSTU34* and *OsABCC15/MRP15*), *OsGSTU34* was upregulated in Wc11 vs. Bc11, Wc18 vs. Bc18, and Wc25 vs. Bc25 with a significant difference, while *OsABCC15* was downregulated. For the other six putative transporters (*OsMATE33, OsMATE39, OsMATE16, OsMATE3, OsMATE7*, and *OsMATE34)*, *OsMATE34 (Os08g0562800*) was upregulated at all levels of comparison, Wc11 vs. Bc11, Wc18 vs. Bc18, and Wc25 vs. Bc25, with a significant difference. *OsMATE3 (Os01g0766000), OsMATE7 (Os02g0821600), OsMATE33 (Os08g0550200), OsMATE39 (Os10g0195000)* were downregulated, and the expressions did not show a significant difference. *OsMATE16 (Os03g0626700)* was upregulated in all comparisons but did not show a significant difference.

We performed the heatmap for these transporters and found that *OsGSTU34 (Os10g0395400)* and *OsMATE34 (Os08g0562800)* were significantly upregulated in black caryopsis and downregulated in white caryopsis whereas *OsMATE3 (Os01g0766000)* and *OsMATE16 (Os03g0626700)* were highly upregulated in black caryopsis 25 DAF but not in other developmental stages. Moreover, these four genes belonged to the same cluster with two different subclusters, subcluster 1 *(OsGSTU34* and *OsMATE34)* and subcluster 2 *(OsMATE3* and *OsMATE16)* (Figure 8B). 

### 3.8. Validation of Transcriptome Data

**Figure 9:**
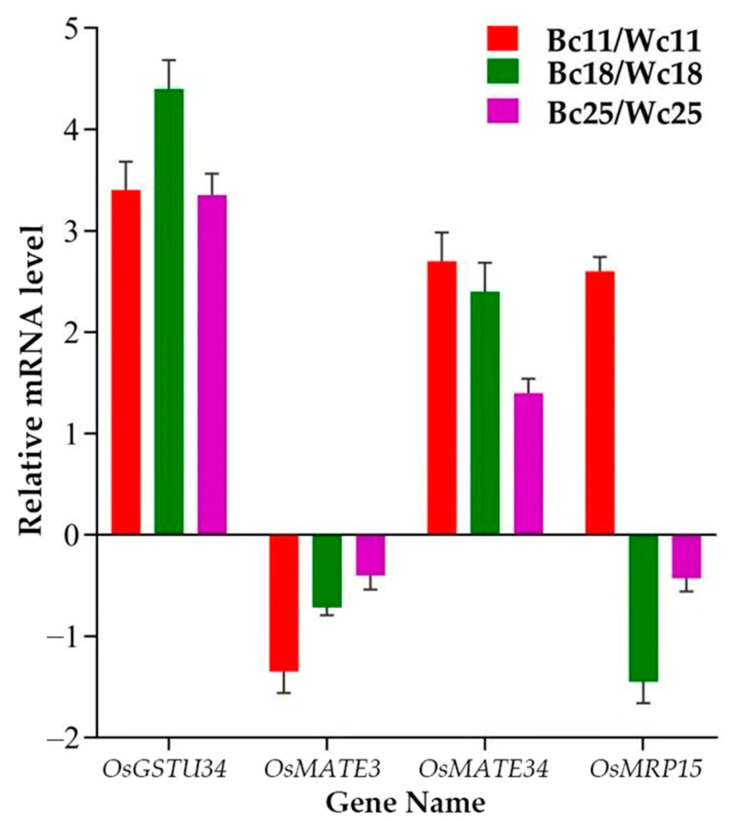
Real-time quantitative PCR validation of transcriptome data for four selected DEGs in all samples. The data were obtained from three independent repeats.

We selected four genes—*OsGSTU34, OsMRP15, OsMATE34*, and *OsMATE3*—to analyze their expression pattern in all samples to validate the transcriptome experiment results (Figure 9). The RT-qPCR results indicated that the selected genes’ expression pattern was consistent with the RNA-seq data having similar expression trends despite the quantitative difference in expression level. 

## 4. Discussion

During the last decade, advances in molecular science have greatly contributed to understanding the function of anthocyanin in the human body [60,61,62] and the plant itself [62,63,64], and the biosynthesis mechanism. Previous studies highlighted that anthocyanins produced on the surface of ER are stored in the tonoplast through two main mechanisms involving different transporters [26,65,66]. These transport mechanisms, compared to xenobiotic transport, ensure the detoxification of cells while playing ecological and physiological benefits and the main molecular actors involved in the transport are GST and tonoplast transporters [31,46,66]. MATE proteins play a noticeable role in rice for detoxification and may be implicated in increasing tolerance of pigmented and some non-pigmented rice against abiotic stress through a metabolite, alkaloid sequestration, hormone, and organic acid transport [67]. In the current study, we combined computational and transcriptome analysis to identify a putative MATE gene involved in anthocyanin transportation in rice caryopsis. The phylogenetic scrutiny is one of the fastest, simplest, and relatively most precise ways to predict gene function. It was reported that in rice and *Arabidopsis* the substrate specificity of most transporter proteins relates to phylogeny [68] and strong evidence has been provided previously in function prediction through phylogenetic analysis. Molecular phylogenetic analysis of MATE proteins was investigated by using the ML algorithm and 1000 bootstraps consensus to represent the evolutionary history. Two MATE proteins—*OsMATE7* and *OsMATE34*—out of 16 selected from the previous study [34] were clustered in the same group with other six well-characterized anthocyanin MATE family including acylated anthocyanin *VvAM1* and *VvAM3* from *V. vinifera* [31,46], flavone glycoside or malonylated anthocyanin *MtMATE2* from *M. truncatula* [47] *SlMATE* or *MTP77* from *S. lycopersicum* [48], *RsMATE8* from *R. sativus* [45], and *AtFFT* from *A. thaliana* [49] associated with glycosylated anthocyanin transportation. Sequence homology showed that *OsMATE34* and *OsMATE7* had the highest identity with *MTP77/SlMATE, MtMATE2, AtFFT, RsMATE8, VvAM1,* and *VvAM3* ranging between 52.66 and 61%. Strikingly, *OsMATE33* and *OsMATE7* exhibited, respectively, 57% and 61.39% identity with *AtFFT* amino sequence associated with anthocyanin transportation in *Arabidopsis thaliana*. This result suggested that *OsMATE7* and *OsMATE34* may have the same function as previously identified AM.

The molecular characterization and structure of *OsMATE7* and *OsMATE34* revealed that the structures of these proteins were different. They were composed of 363 and 489 amino acid residues with a molecular weight of 38.74 and 51.6 kDa, respectively. The three-dimensional protein configuration showed a difference with *OsMATE7* but a remarkably high similarity between *OsMATE34* and the anthocyanin MATE-like proteins from homolog plants (Figure S4). The prediction of the transmembrane domain showed that unlike most AM including *OsMATE34* that had 10–12 segments, *OsMATE7* had only six segments. A previous study reported that the MATE family consisted of a unique topology predicted to have 10–12 TM helices with long, cytoplasmatic C and N termini [59]. Protein motif structure showed a complete difference between *OsMATE7* with only eight motifs compared to other AM of the same clade including *OsMATE34* that were 11 motifs. The physicochemical properties revealed that isoelectric point, aliphatic index, instability index, and GRAVY of *VvAM1, VvAM3, MtMATE2, SlMATE/MTP77, RsMATE8*, and *AtFFT* were similar and the values were very close to *OsMATE34* relatively high compared to *OsMATE7* with a lower value. This noticeable difference in MATE protein structures and properties, particularly *OsMATE7* to *OsMATE34* and other anthoMATEs, might be due to functional diversities that occurred during the evolution and suggested that *OsMATE34* and *OsMATE7* may have slightly different functions and expression pattern. Although *VvAM1, VvAM3, MtMATE2, SlMATE/MTP77, RsMATE8, AtFFT, OsMATE7,* and *OsMATE34* belong to the same cluster, *OsMATE34* was very close to AM from other plants regarding the molecular and structural analysis. Thus, it can be inferred that *OsMATE34* is a putative anthocyanin transporter in rice and may have the same function as the other well-characterized AM reported in different plants.

To confirm the role of *OsMATE34*, we carried out transcriptome analysis of black and white rice caryopsis at different developmental stages. The result revealed differential expression of anthocyanin biosynthesis genes. Although the two cultivars were black rice, the difference between them relied on the caryopsis color mainly their pericarp color. Caryopsis is the most important part of rice as a staple food. The pairwise comparison showed that the number of DEGs was higher in black caryopsis than white caryopsis and the anthocyanin biosynthesis genes were upregulated in black rice with high expression observed 18 DAF. Meanwhile, the quantification of anthocyanin in black rice caryopsis revealed that the concentration of the two majors rice anthocyanin C3G and P3G attained their peaks at 18 DAF (Figure S1) and started to decrease after this period. This result demonstrated a positive correlation between gene expression peculiarly *OsMATE34* and the accumulation of anthocyanin in the rice caryopsis. Our results were consistent with the previous studies reporting that in black rice caryopsis, anthocyanin was found in the bran in high amounts and accumulates progressively mainly inside the pericarp from 7 DAF, then inside the testa and aleurone layer after 15–30 DAF [7,15]. Likewise, the expression of all anthocyanin related-genes was upregulated and attained their peak after about 13 DAF [69]. In the current experiment, most of these genes and transcriptome factors mainly Kala4 coding for bHLH transcriptome factors, Kala3 and OsC1 coding for Myb transcriptome factor, and WD40 were upregulated and the log2FC of Bc vs. Wc showed that the difference was greatly significant during these three periods (Table S6). This result is in line with other studies showing that the structural genes for anthocyanin accumulation in black rice (*OsPAL, OsCHS, OsCHI, OsF3H, OsDFR, OsANS/LDOX*, and *ANS OsUFGT/Os3GT)* and *OsADR* function as a single unit activated by a transcription factor complex MYB-bHLH-WD40 (MBW) [69,70,71,72]. GO and KEGG enrichment indicated that the flavonoid metabolic process (GO:0009812) was the predominant biological process, and most of these activities were expressed in the vacuole and aleurone grain, suggesting that most processes at these stages of development occurred inside the vacuole and in the aleurone layer. These results corroborated with some previous studies showing that anthocyanin accumulated in the aleurone layer of caryopsis [15,73] and the vacuole of the cells [65,66]. The metabolism pathways and flavonoid biosynthesis pathways (KEGG: map 00941) were particularly enriched. It was reported that anthocyanin and proanthocyanins are synthesized through the flavonoid biosynthesis pathway [74,75], suggesting that anthocyanin biosynthesis was the most predominant physiological activity at these growing and developmental periods. We reasonably speculated that these various physiological activities and the flavonoid biosynthesis genes governed the metabolic differences that have been observed and would have influenced DEGs.

The eight transporter genes (*OsMRP15, OsGSTU34, OsMATE33, OsMATE39, OsMATE16, OsMATE3, OsMATE7*, and *OsMATE34)* investigated in this transcriptome analysis showed different expression pattern. *OsGSTU34* and *OsMATE34* were significantly upregulated at all stages (11 DAF, 18 DAF, and 25 DAF) and the result indicated that the difference in their expression was significant between black caryopsis and white caryopsis. For both genes, the highest expressions were observed 18 DAF in black caryopsis and decreasing at 25 DAF. This result was consistent with the RT-qPCR (Figure 9). The differential expression clusters analysis showed that *OsGSTU34* and *OsMATE34* had the same expression pattern and cluster with nine main anthocyanin biosynthesis genes that were also upregulated in this study (Figure 8B). In a previous study, Ma and colleagues [32] elucidated the role of *OsGSTU34* genes encoding glutathione-s-transferase and *OsMRP15* in anthocyanin accumulation through CRISPR/Cas 9 genome targeting, an induced mutation of *OsGSTU34* and *OsMRP15* results in the loss of function of purple leaves coloration. This result indicated the role of *OsGSTU34* and *MRP15* in anthocyanin biosynthesis. As reported by Martinoia and co-authors [76,77], ABC-type transporters recognized and sequestered the glutathione conjugates to the vacuoles or exported them to the cell wall. However, our results indicated that the expression of both *OsGSTU34* and *OsMRP15* was completely different and *OsGSTU34* was upregulated in black caryopsis but not in white caryopsis. Unlike *OsGSTU34, OsMRP15* was downregulated in black and white caryopsis, suggesting that the *OsMRP15* gene may not be expressed in the caryopsis as it does in purple leaves and that the functions of both genes are not strictly linked. The function of *OsMRP15* in the transport of anthocyanin in rice leaves would be taken over by *OsMATE34* in rice caryopsis as the MATE transporters possess a membrane transport function. Moreover, *OsGSTU34* and *OsMATE34* had the same expression pattern at all developmental stages. Taken together, it is obvious to say that *OsMATE34* may have the same function as *OsGSTU34* and plays the central role in the sequestration of anthocyanin inside the vacuole. Consequently, it could be involved in the anthocyanin transportation in rice, mainly in rice caryopsis.

*OsMATE34* is a gene consisting of three exons and two introns. Its protein sequence is a long chain of 489 amino acids with a molecular weight of 51.6 kDa; 12 TM was topologically revealed, and the secondary structure predicted α-helix, extended strand, β-turn, and random coil, helices that were identified with a functional opening to achieve the function of transport of molecule. This conforms with Yazaki and co-workers [78] reporting on the primary feature of the MATE family. The function and properties of proteins essentially rely on their physiochemical properties and proteins with good properties can sustain unfavorable conditions and function properly. *OsMATE34* has the same characteristics as anthocyanin Mate-like proteins. In silico analysis showed that the computing value of the PI was 5.6 similar to *MtMATE2* suggesting that this protein is acidic. Likewise, AI was very close to that of *VvAM3* with, respectively, 116.93 and 116.06. This value is greater than the reference value revealed that *OsMATE34* is highly thermostable. It was reported that AI of a globular protein determines their thermostability; values greater than 65 are considered thermostable, and the higher the AI, the more stable the proteins are [79]. Moreover, the I.I range to 32.98 similar to that of *AtFFT* (33.84). This suggested that it can chaperone plants, maintaining the physiological function, and protecting against specific cellular stress that can lead to protein denaturation. The solubility of *OsMATE34* was analyzed through the GRAVY.to predict water and protein interaction, also qualified and quantified the hydrophobic and hydrophilic property of the protein. A positive value indicates hydrophobic protein. Our results indicated that this protein is hydrophobic, similar to other anthocyanin transporters of MATE type, and the score ranges to 0.746, the same as with *AtTT12*. These similarities found between *OsMATE34* and the previous AM can presume similar substrates, thus the same function can be inferred. From the current study, it can be seen that AM proteins are acidic, highly stable, and hydrophobic.

The basic information about MATE protein localization predicted that *OsMATE34* is localized to the plasma membrane and in the tonoplast. This result corroborated with the previous studies showing that *VvAM1, VvAM2,* and *VvMATE1*, responsible for anthocyanin transportation in grapevines, were localized in the tonoplast [31,52]. Besides, the function of the transporter gene is determined by its subcellular localization [57]. It was reported that the MATE proteins are mainly localized on either the plasma membrane or vacuolar membrane. When located to the plasma membrane, it participates to exit the substrate out of the cell in exchange for H+, while when located to the vacuolar membrane, it enhances the uptake of the substrate due to the pH gradient which is basic in the cytoplasm (7–7.4) and acid in the vacuole (5.5) [80]. The MATE proteins are a component of flavonoid biosynthetic pathway transporters that combine the transport of intended molecules across an H+ or Na+ electrochemical gradient of ions [78]. The possible localization of *OsMATE34* to the vacuolar membrane suggested its implication in the transportation of anthocyanin inside the vacuolar lumen. *OsMATE34* was reported as a homolog of *AtTT12* found across vacuolar membrane mediating the membrane transport of C3G and C3G derivatives [81], which generally occupied approximately 65–90% of anthocyanin found in rice and 90% in the cultivar used in this study. Similarly, *MTP77*, a homolog of *AtTT12*, was identified in the vacuole as a putative anthocyanin transporter in tomato [48]. Like most secondary metabolites, it is necessary for anthocyanins produced in the cytoplasm to be delivered into the vacuole because they may be toxic to the plant. Therefore, to avoid harmful effects and improve the efficiency of their production, they need to be sequestrated in the vacuolar lumen [82,83]. The MATE protein family, peculiarly *OsMATE34* along with *OsGSTU34*, may play a central role for anthocyanin sequestration in the vacuole of rice caryopsis cells.

## 5. Conclusions

This study aimed to identify and characterize the MATE transporter involved in anthocyanin transportation in rice, mainly in rice caryopsis. Based on the computational and transcriptome results, we suggested that *OsMATE34 (Os08g0562800)* along with *OsGSTU34 (Os10g0395400)* were involved in anthocyanin transportation in black rice caryopsis. *OsMATE34* was reported as a homolog of *AtTT12* and *AtFFT* found across the vacuolar membrane mediating the membrane transport of C3G, which is the main anthocyanin found in rice caryopsis. Protein localization predicted that *OsMATE34* was localized in the plasma membrane and vacuole. Thus, it can be reasonably inferred that *OsMATE34* plays a central role in the anthocyanin sequestration in the vacuole of rice caryopsis cells. As transporters are the major players in the trafficking of anthocyanins, this finding is a clue to enhance the accumulation of anthocyanin in rice caryopsis and the endosperm. This study is the first to highlight MATE-type transporters in the accumulation of anthocyanins in rice caryopsis. However, a further study is currently being carried out to thoroughly understand by which transport mechanism *OsMATE34* is involved in anthocyanin transportation and the possible interaction with GST transporters.

## Figures and Tables

**Table 1:** Anthocyanin content (µg. g −1 FW) in black and white caryopsis during the development stages.

Type of Anthocyanin	Rice Developmental Stages							
	Milk (11 DAF)		Dough (18 DAF)		Mature (25 DAF)		Fully Ripe (35 DAF)	
	Wc11	Bc11	Wc18	Bc18	Wc25	Bc25	Wc35	Bc35
Pt3G	ND	ND	ND	ND	ND	ND	ND	ND
P3G	ND	163.75 ± 5	ND	295.33 ± 4	ND	97.52 ± 10	ND	35.98 ± 7
C3G	ND	1600.96 ± 15	ND	4206.91 ± 18	ND	1212.87 ± 10	ND	814.59 ± 5.5

Anthocyanin content is represented as value ± SD of three biological replicates; ND, Not detected; Pt3G, petunidin-3-O-glucoside; P3G, peonidin-3-O-glucoside; C3G, cyanidin-3-O-glucoside; DAF, days after flowering.

**Table 2:** Summary of RNA seq data and reads mapping.

Sample ID	Raw Reads	Clean Reads	Total Mapped Reads	Q20 (%)	Q30 (%)	GC (%)
Wc11-rep1	46234788	45716764	42690828 (93.38%)	98.81	96.31	51.99
Wc11-rep2	47796190	47120470	43973125 (93.32%)	98.74	96.12	51.79
Wc18-rep1	53602144	53141526	48976170 (92.16%)	98.69	95.89	52.4
Wc18-rep2	51253398	50855026	47207849 (92.83%)	98.83	96.28	52.22
Wc25-rep1	53062226	52561118	48421904 (92.12%)	98.74	96.16	54.88
Wc25-rep2	56203098	55792292	51481571 (92.27%)	98.87	96.4	53.63
Bc11-rep1	44487444	43814814	41492963 (94.7%)	98.79	96.27	51.4
Bc11-rep2	47527712	46925570	44457493 (94.74%)	98.78	96.17	51.67
Bc18-rep1	54482624	54089936	51460136 (95.14%)	98.8	96.21	52.01
Bc18-rep2	54430818	53880872	51159662 (94.95%)	98.71	95.99	52.25
Bc25-rep1	57726156	57135956	54078567 (94.65%)	98.78	96.23	54.57
Bc25-rep2	49975758	49497564	46688293 (94.32%)	98.78	96.21	54.56

Rep1 and Rep2 indicate respectively replication 1 and replication 2. Wc11 = White caryopsis, 11 days after flowering; Wc18 = White caryopsis, 18 days after flowering; Wc25 = White caryopsis, 25 days after flowering; Bc11 = Black caryopsis, 11 days after flowering; Bc18 = Black caryopsis, 18 days after flowering; Bc25 = Black caryopsis, 25 days after flowering; GC%, represents Guanine-Cytosine content which is often rich in the coding region and calculated as count(G + C)/count(A + T + G + C) * 100%; Q20 indicates the probability of an incorrect base call is 1 in 100; Q30, 1 in 1000 and their value refer respectively to the percentage of bases with sequencing quality above 99% and 99.9%.

## Data Availability

Raw data can be provided to researchers on request to the corresponding or first author.

## Supplementary Materials

The following are available online at https://www.mdpi.com/article/10.3390/genes12040583/s1. Table S1: Primers used for RT-qPCR analysis; Table S2: Physiochemical property of MATE proteins; Table S3: The differentially conserved motif of MATE gene family; Table S4: Similarity index and identity; Table S5: The basic information about MATE protein localization; Table S6: Expression pattern of anthocyanin biosynthesis and transporter genes based on fold-change levels in Bc and Wc; Figure S1: Anthocyanin quantification in black rice caryopsis at different developmental stages; Figure S2: Secondary structures of the 6 rice MATEs proteins and 7 anthocyanin MATEs-like proteins predicted by SOPMA; Figure S3: Topological analysis of OsMATEs; Figure S4: Comparison of the three-dimensional configuration of two putative OsMATEs and six anthoMATEs. File S1: GO and KEGG pathway analysis; File S2: Differentially expressed genes of anthocyanin biosynthesis pathway analysis and function; File S3: Quality control protocol of RNA-sequencing analysis.

## Abbreviations

**AI**	Aliphatic Index
**ABC**	ATP-Binding Cassette
**AM**	AnthoMATE
**AVI**	Anthocyanic Vacuolar Inclusion
**bHLH**	Basic helix-loop-helix
**Bc**	Black Caryopsis
**C3G**	Cyanidin-3-O-Glucoside
**DEGs**	Differentially Expressed Genes
**DAF**	Days After Flowering
**ER**	Endoplasmic Reticulum
**FDR**	False Discovery Rate
**FW**	Fresh Weight
**GST**	Glutathione-S-Transferase
**GO**	Gene Ontology
**GRAVY**	GRAND Average of Hydropathicity Index
**HMMTOP**	Hidden Markov Model Topology
**HPLC**	High-Performance Liquid Chromatography
**I.I**	Instability Index
**JTT**	Jones–Taylor–Thornton
**KALA**	Key Activator Loci for Anthocyanin
**KEGG**	Kyoto Encyclopedia of Genes and Genomes
**LT**	Ligandin Transportations
**MATE**	Multidrug and Toxic Compound Extrusion
**MEME**	Multiple Expectation Maximization for Motif Elicitation
**ML**	Maximum Likelihood
**MRP**	Multidrug Resistance-associated Proteins
**MW**	Molecular Weight
**P3G**	Peonidin-3-O-Glucoside
**PVC**	Pre-vacuolar Compartment
**RSEM**	RNA sequence by expectation maximization
**ROS**	Reactive Oxygen Species
**SOPMA**	Self-Optimized Prediction Method from Alignment
**TAC**	Total Anthocyanin Content
**TMHMM**	Transmembrane Helices Hidden Markov Model
**TPM**	Transcripts Per Million
**VT**	Vesicular Transport
**Wc**	White Caryopsis
