# Supplementary material for: Computational and Transcriptomic Analysis Unraveled OsMATE34 as a Putative Anthocyanin Transporter in Black Rice (Oryza sativa L.) Caryopsis
Source: Genes (Basel). 2021 Apr 16;12(4):583. doi: 10.3390/genes12040583 (PMC8073145; doi:10.3390/genes12040583)
Supplement: Supplementary file 1 [file genes-12-00583-s001.zip › genes supplementary Table and Figure.pdf]

Table S1. Primers used for RT-qPCR analysis.

Gene	transcript ID	Forward primer	Reverse primer
OsMRP15	*OS06T0158900*	GATACAATCTAGACCCCCTTGG	CAACAAGTGAATCCAATCCCTG
OsGSTU34	*OS10T0395400*	GATGAGCCCGTTCACGAT	AACTTGTCATCTATGTAGGCGG
OsMATE3	*OS01T0766000*	TGGCTTGTCTCTGCTCTATATG	GATGAGGAACTGGAAGATCTCC
OsMATE34	*OS08T0562800*	GAGTTGCACTTCAAACACTCAT	CTCCTTGTAAGAGAGGTTTGGT
OsActin-1	*Os03T0718100*	GAGTATGATGAGTCGGGTCCAG	ACACCAACAATCCCAAACAGAG

Table S2. Physiochemical property of MATE proteins.

Plant species	gene	Accession No / ID /AGI code	Chr	Exon count	MW (KDa)	A.A	PI	AI	II	GRAVY
Vitis vinifera	VvMate1	XP_002282932.1	12	8	54.90	504	8.38	127.72	24.67	0.809
Vitis vinifera	VvMATE2	XP_002282907.1	12	8	54.41	506	6.99	133.95	23.91	0.887
Medicago truncatula	MtMATE1	ACX37118.1	5	9	54.89	504	6.50	128.85	23.53	0.859
Brassica rapa	BrTT12	ACJ36213.1	7	8	55.17	507	8.36	124.02	28.40	0.752
Arabidopsis thaliana	AtTT12	At3g59030.1	3	8	55.15	507	8.36	120.18	30.21	0.745
Fragaria ananassa	FaTT12	AUA60209.1			55.01	506	5.51	126.01	27.69	0.806
Malus domestica	MdMATE1	ADO22709.1/ GU064953.1	9	7	55.12	505	6.52	125.70	25.28	0.799
Malus domestica	MdMATE2	ADO22711.1/ GU064955	17	7	55.97	514	6.99	123.72	24.37	0.761
Oryza sativa	OsMATE55	LOC_Os12g42130.1	12	8	54.10	500	6.32	128.36	25.35	0.848
Oryza sativa	OsMATE15	LOC_Os03g37640.1	3	7	53.62	500	6.52	119	32.30	0.763
Oryza sativa	OsMATE13	LOC_Os03g37470.1	3	6	37.35	350	7.52	120.97	23.60	0.881
Raphanus sativus	RsMATE2	MF360948	5	7	55.04	505	5.18	117.80	27.62	0.708
Oryza sativa	OsMATE14	LOC_Os03g37490.1	3	7	55.79	520	5.24	114.21	30.58	0.731
Oryza sativa	OsMATE12	LOC_Os03g37411.1	3	7	53.84	500	6.98	114.52	30.51	0.702
Oryza sativa	OsMATE34	LOC_Os08g44870.1	8	3	51.60	489	5.6	116.93	32.79	0.746
Oryza sativa	OsMATE7	LOC_Os02g57570.1	2	2	38.74	363	4.72	110.06	32.98	0.528
Solanum lycopersecum	Slmate /MTP77	AAQ55183.1	3	7	55.17	506	5.89	119.13	21.64	0.650
Medicago truncatula	MtMATE2	ADV04045.1	1	7	54.52	501	5.68	126.45	27.82	0.785
Arabidopsis thaliana	AtFFT	At4g25640	4	7	53.22	488	6.13	120.88	33.84	0.798
Raphanus sativus	RsMATE8	MF360954	8		53.37	490	6.89	119.80	31.51	0.792
Vitis vinifera	VvAM3	ACN91542.1	16	6	53.85	493	6.53	116.06	28.46	0.678
Vitis vinifera	VvAM1	ACN88706.1	16	7	53.50	489	5.89	118.12	24.07	0.725
Oryza sativa	OsMATE3	LOC_Os01g56050.1	1	8	51.58	484	9.58	119.94	30.64	0.728
Oryza sativa	OsMATE16	LOC_Os03g42830.1	3	8	51.83	477	8.93	117.80	26.65	0.76
Oryza sativa	OsMATE39	LOC_Os10g11860.1	10	8	50.22	464	8.82	117.16	30.09	0.806
Raphanus sativus	RsMATE3	MF360949	3		56.48	519	5.34	112.76	27.96	0.812
Oryza sativa	OsMATE33	LOC_Os08g43654.1	8	3	56.17	522	6.71	116.07	37.06	0.698
Oryza sativa	OsMATE9	LOC_Os03g08900.1	3	8	52.96	489	6.97	117.89	26.69	0.775
Oryza sativa	OsMATE31	LOC_Os08g37432.1	8	8	52.55	489	6.09	123.25	22.92	0.802
Oryza sativa	OsMATE35	LOC_Os09g29284.1	9	8	51.97	482	9	117.97	40.07	0.730
Oryza sativa	OsMATE48	LOC_Os11g03484.1	11	4	36.14	329	5.09	114.98	38.61	0.652
Oryza sativa	OsMATE51	LOC_Os12g03200.1	12	6	33.89	311	5.08	116.62	39.96	0.648
Oryza sativa	OsMATE47	LOC_Os11g03240.1	11	8	54.68	497	6.36	117.89	38.93	0.689
Oryza sativa	OsMATE 52	LOC_Os12g03230.1	12	7	54.68	497	6.36	117	38.93	0.689
Oryza sativa	OsMATE49	LOC_Os11g03500.1	11	9	54.24	495	6.33	115.78	32.01	0.606
Oryza sativa	OsMATE53	LOC_Os12g03260.1	12	8	55.44	507	6.01	116.13	30.83	0.615
Arabidopsis thaliana	AtFRD3	At3g08040	3	12	55.95	526	9.63	114.70	29.83	0.613
Raphanus sativus	RsMATE7	MF360953	7		48.11	451	5.9	117.07	36.64	0.598

The MATE transporter genes were denominated systematically based on their physical locations on the chromosomes of rice. The MW (Molecular weight in kiloDaltons), A.A (Number of amino acids), PI (isoelectric point), AI (aliphatic index), II (instability index), and GRAVY (GRAND average of Hydropathicity Index) were computed using the ExPASy Compute pI/ Mw tool (http:// https://web.expasy.org/cgi-bin/protparam; accessed on 30 August 2020).

Table S3: The Differentially conserved motif of MATE gene family

MOTIF	PROTEIN SEQUENCE	LENGHT
**MEME-1**	PLLAITILLNSIQPVLSGVAVGSGWQALVAYVNLGCYYJIGJPJGCLLGF	50
**MEME-2**	GFAYGIMLGMASALETLCGQAYGAKQYHMLGVYLQRS	37
**MEME-3**	CKETWTGFSWKAFTGJWAFVKLSLASAVMLCLEJWYNQILVLJTGLLKNA	50
**MEME-4**	EIALDSJSICMNINGWEMMISLGFNAAISVRVSNELGAGHPRAAKFSVFV	50
**MEME-5**	KGIWWGMIIGTLLQTLILLFJTARTBWNKEVEEASERLKKW	41
**MEME-6**	IAAAAGTFALGLIPQJFAYAINFPLQKFLQAQSKVNPLAYI	41
**MEME-7**	YGLSFVTQAFVGHJGALELAAASJASTVIQ	30
**MEME-8**	JLLPJYWFATPJLIALGQSPE	21
**MEME-9**	RDYLAYJFTSDKEVIDAVSDL	21
**MEME-10**	HVLLSWLVVYVLGLGLLGAAL	21
**MEME-11**	RLVAWESKKLWRIAGPSIFVS	21
**MEME-12**	TLSFSWWJJVAAQLAYIVFSP	22

Table S4. Similarity index and identity.

OsMATE	OTHER MATE	Similarity index	IDENTITY
OsMATE 34	MTP77/ SlMATE	51	52.74
	MtMATE2	51	52.67
	AtFFT	55	56.99
	RsMATE8	54	55.21
	VvAM3	54	54.56
	VvAM1	54	52.51
	RsMATE3	42	44.77
OsMATE 7	MTP77	41	59.68
	MtMATE2	39	56.23
	AtFFT	42	61.39
	RsMATE8	42	60.66
	VvAM3	39	57.19
	VvAM1	39	57.52
	RsMATE3	31	43.41
OsMATE33	RsMATE3	50	50.97
	MTP77/ SlMATE	44	46.04
	MtMATE2	46	47.37
	AtFFT	44	47.63
	RsMATE8	45	47.23
	VvAM3	44	45.92
	VvAM1	44	46.31
OsMATE39	RsMATE3	46	47.08
	MTP77/ SlMATE	41	41.34
	MtMATE2	41	41.99
	AtFFT	40	41.34
	RsMATE8	40	40.69
	VvAM3	41	41.34
	VvAM1	41	40.91
OsMATE16	RsMATE3	51	50.22
	MTP77/ SlMATE	44	44.73
	MtMATE2	45	45.49
	AtFFT	46	47.20
	RsMATE8	46	46.34
	VvAM3	46	46.02
	VvAM1	46	45.24
OsMATE 3	RsMATE3	47	51.58
	MTP77/ SlMATE	44	46.51
	MtMATE2	42	45.03
	AtFFT	43	47.45
	RsMATE8	44	46.07
	VvAM3	43	45.24
	VvAM1	43	44.26

Sequence identity was analyzed using Clustal Omega multiple sequence analysis https://www.ebi.ac.uk/Tools/msa/clustalo/; accessed on 21 August 2020

Table S5: The basic information about MATE protein localization.

Proteins	plas	vacu	ER	GOLG	Cyto	chlo	mito
VvMate1 T12	6	4		3	1		
VvMATE2	2	9		3			
MtMATE1	7	5	1	1			
BrTT12	8	3	1	2			
AtTT12	6	4	1	3			
FaTT12	6	5	1	2			
MdMATE1	6	5		3			
MdMATE2	6	4	1	3			
OsMATE55	7	4	1	2			
OsMATE15	6	4	2	2			
OsMATE13	4	10					
RsMATE2	7	4	1	2			
OsMATE14	6	4	1	2	1		
OsMATE12	8	3	2	1			
OsMATE34	6	5	1	1	1		
OsMATE7	6.5	1	4		4	1	1
SlMATE/MTP77	9	4	1				
MtMATE2	9	4	1				
AtFFT	13	1					
RsMATE8	8	2	1	3			
VvAM3	6	4	1	3			
VvAM1	9	2	1	2			
OSMATE3	11	1	1			1	
OSMATE16	1	10		2	1		
OSMATE39	6	6		1	1		
RsMATE3	7	4		3			
OsMATE33	12	2					
OSMATE9	9	4	1				
OsMATE31	11	1	2				
OSMATE35	3	6	1	3	1		
OSMATE48	10	1	2		1		
OSMATE51	9	2	2		1		
OsMATE47	11	1	2				
OsMATE52	11	1	2				
OsMATE49	11	1	2				
OsMATE53	13	1					
AtFRD3	3	8		2	1		
RsMATE7	5	4	3			2	

Subcellular localization prediction using https://wolfpsort.hgc.jp/; accessed on 8 September 2021. plas, vacu, ER, Golg, Cyto,Chlo, mito represent respectively plasma membrane protein, vacuole, endoplasmic recticulum, golgi apparatus, chloroplast and mitochondria.

Table S6: Expression pattern of anthocyanin biosynthesis and transporter genes based on fold-change levels in Bc and Wc.

gene groups	gene_ID	Gene name	Log2FC Bc11/Wc11	Log2FC Bc18/Wc18	Log2FC Bc25/Wc25
**Transporter genes**	Os06g0158900	OsABCC15/ MRP15	5.29***	-1.19***	-0.35*
	Os10g0395400	OsGSTU34/GST	9.37***	12.47***	9.57***
	Os01g0766000	OsMate3	-1.49*	-0.78*	-0.32*
	Os02g0821600	Mate7	-0.95*	0.00	0.00
	Os03g0626700	Mate16	4.29*	0.57*	0.55*
	Os08g0550200	Mate 33	0.00	-3.97*	-2.28*
	Os08g0562800	Mate 34	9.21***	6.43***	3.98***
	Os10g0195000	OsSTA240/ MATE39	0.00	-3.23*	-3.81***
**Phenylalanine pathway Structural genes**	*Os02g0627100*	*OsPAL1/ OsPAL*	7.12 ***	2.81 ***	2.01 ***
	Os05g0320700	*C4H2/ OsC4H*	2.90 ***	2.99 ***	1.24 *
	Os02g0177600	*4CL3/Os4CL*	3.78 ***	2.49 ***	1.30 *
	Os11g0530600	*CHS/ OsCHS*	9.20 ***	9.33 ***	7.21 ***
	Os03g0819600	*ghl/ OsCHI*	4.58 ***	2.23 ***	1.76 ***
**Anthocyanin pathway Structural genes**	*Os04g0662600*	*OsF3H-1/OsF3H*	7.37 ***	8.71 ***	7.16 ***
	Os10g0320100	*OsF3'H*	8.09 ***	8.38 ***	7.59 ***
	Os03g0367200	*OsCYP75A11/ OsF3'5'H*	0.00	2.17 *	4.15 *
	Os01g0633500	*Rd/OsDFR*	9.49 ***	11.65 ***	11.76 ***
	Os01g0372500	*OsANS*	10.06 ***	11.58 ***	5.59 ***
	Os03g0259400	OsLAR	5.75 *	2.43 *	3.20 ***
	Os04g0630800	*OsANR*	-0.05 *	1.18 *	2.38 ***
**Decorating genes**	*Os06g0192100*	*UGT/Os3GT*	3.62 ***	5.25***	2.44 ***
	Os08g0157500	*ROMT9/Os3'MT*	0.57 *	0.05 *	0.63 *
	Os04g0557800	*Pl/OSB1*	0.00	-4.04 *	-2.23 *
	Os04g0557500	*Kala4/OSB2*	8.65 ***	10.59 ***	9.98 ***
**Regulatory genes**	*Os06g0205100*	*C/OsC1*	0.00	0.00	-1.23 *
	Os03g0410000	*OsMyb3/Kala 3*	3.94 *	5.06 ***	4.18 ***
	Os02g0682500	*OsWD40-50 /OsWD40*	0.99 *	3.69 ***	2.26 ***

(***) The difference is significant between black and white caryopsis; (*) the difference is not significant between black and white caryopsis; The fold change with positive and negative values represents upregulated and downregulated genes.

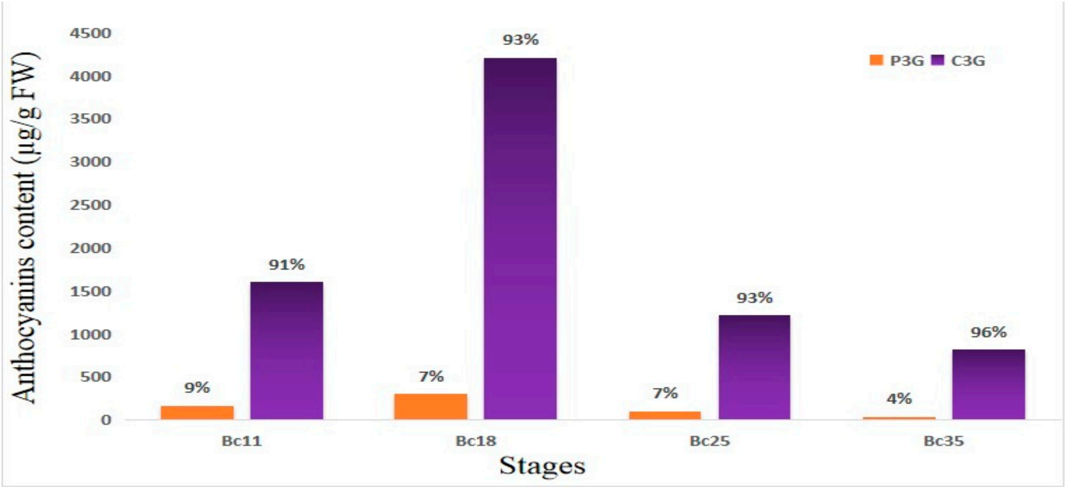

Figure S1. Anthocyanin quantification in black rice caryopsis at different stages; FW, fresh weight; Bc, Black caryopsis; the numbers after Bc in the graph represent days after flowering; P3G, Peonidin-3-O-glucoside in orange bar; C3G, cyanidin-3-O-glucoside; DAF, days after flowering; the number above each bar represents the relative percentage of C3G and P3G in TAC.

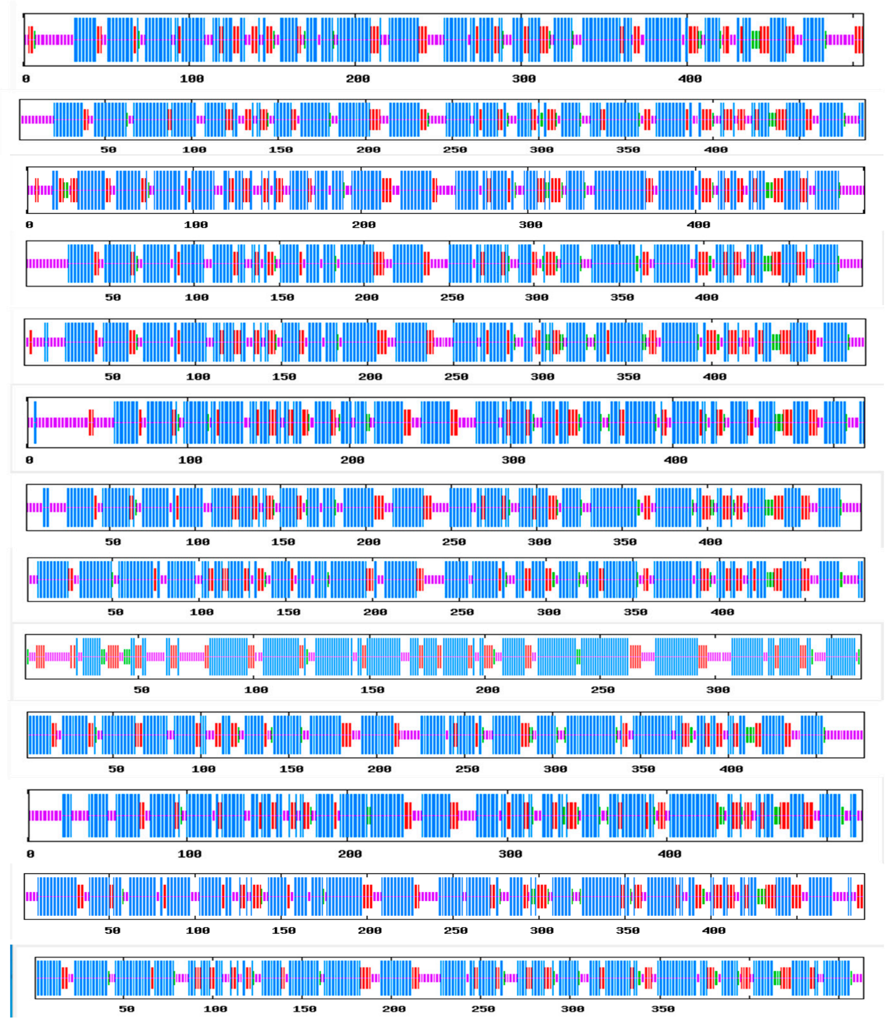

Figure S2. Secondary structures of the 6 rice MATEs proteins and 7 anthocyanin MATEs-like proteins predicted by SOPMA. The α-helix, extended strand, β-turn, and random coil are denoted as the longest (blue), medium long (dark red), short(green), and the shortest (pink) vertical bars, respectively.

ne ermined

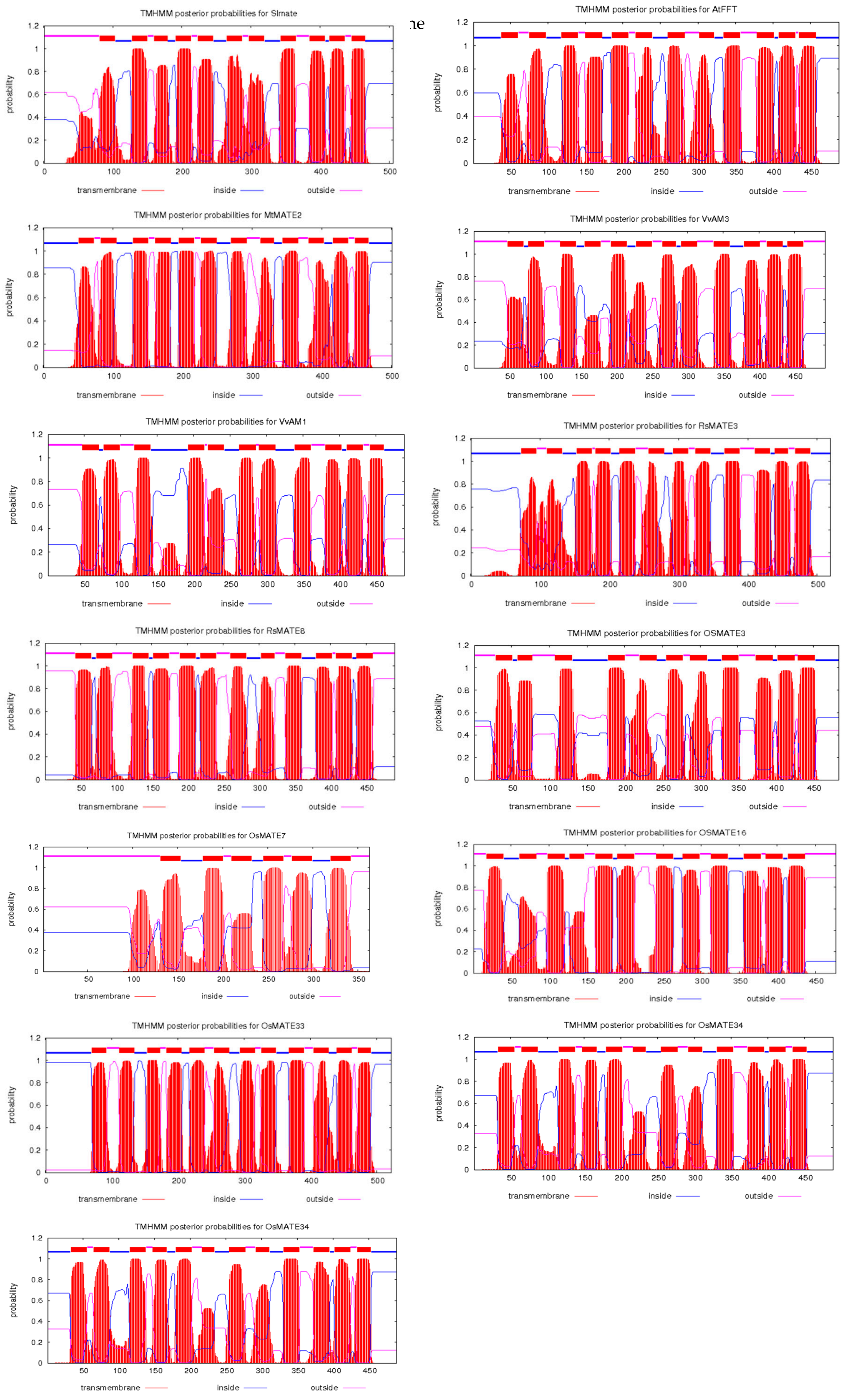
TMHMM posterior probabilities for Slmate
TMHMM posterior probabilities for AtFFT
TMHMM posterior probabilities for MtMATE2
TMHMM posterior probabilities for VvAM3
TMHMM posterior probabilities for VvAM1
TMHMM posterior probabilities for RsMATE3
TMHMM posterior probabilities for RsMATE8
TMHMM posterior probabilities for OSMATE3
TMHMM posterior probabilities for OsMATE7
TMHMM posterior probabilities for OSMATE16
TMHMM posterior probabilities for OsMATE33
TMHMM posterior probabilities for OsMATE34
TMHMM posterior probabilities for OsMATE34
probability
transmembrane
inside
outside

using the TMHMM2 program.

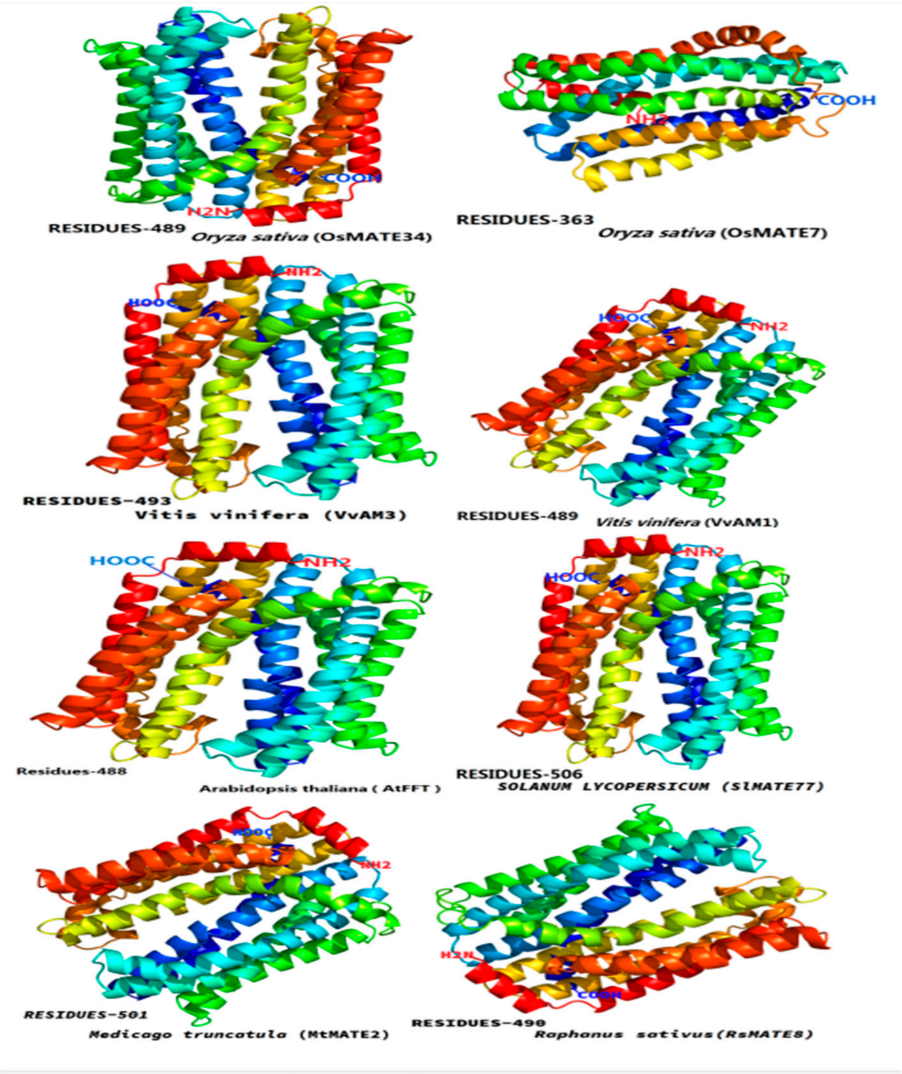

Figure S4. Comparison of the three-dimensional configuration of two putative OsMATEs and six anthoMATEs.
