# Supplementary material for: Computational and Transcriptomic Analysis Unraveled OsMATE34 as a Putative Anthocyanin Transporter in Black Rice (Oryza sativa L.) Caryopsis
Source: Genes (Basel). 2021 Apr 16;12(4):583. doi: 10.3390/genes12040583 (PMC8073145; doi:10.3390/genes12040583)
Supplement: Supplementary file 1 [file genes-12-00583-s001.zip › Supplementary file 3.pdf]

**Quality control protocol of RNA-SEQ data**

Since the original sequencing data contained sequencing adapter sequences, low-quality reads, sequences with higher N (N represents uncertain base information), and sequences with too short length, these may seriously affect the quality of subsequent assembly. As a guarantee for the accuracy of subsequent bioinformatics analysis, the original sequencing data was first filtered to obtain high-quality sequencing data (clean data) to ensure the smooth progress of subsequent analysis. High-quality reads were assembled using SeqPrep (http://github.com/jstjohn/SeqPrep) and Sickle (http://github.com/najoshi/sickle) software with default parameters. The specific steps and sequence were as follows:

1) Removing reads linker sequence, due to the removal from the joint as a result even without inserted fragment reads;

2) Trim off the bases with low quality (quality value less within last 30) at the end of the sequence (3'End). If there are still bases with a quality value less within last 10 in the remaining sequence, the entire sequence was removed;

3) The removal of N ratio exceeds 10% of the reads;

4) The adaptor and the sequence whose length was less than 50bp after quality trimming was discarded.

After the quality cut is completed, statistics and quality evaluation of the quality control data were performed again, including (1) A/T/G/C Base content distribution statistics; (2) base quality distribution statistics ; (3) Base error rate distribution statistics.

Connector information was added .

5'AGATCGGAAGAGCACACGTC

3'AGATCGGAAGAGCGTCGTGT
